# 2,4-Di-*tert*-butyl-6-[1-(3,5-di-*tert*-butyl-2-hydroxy­phen­yl)eth­yl]phenyl 4-methyl­benzene­sulfonate

**DOI:** 10.1107/S1600536808042323

**Published:** 2008-12-17

**Authors:** Jincai Wu, Xiaobo Pan, Lei Wang, Lihui Yao

**Affiliations:** a College of Chemistry and Chemical Engineering, State Key Laboratory of Applied Organic Chemistry, Lanzhou University, Lanzhou 730000, People’s Republic of China

## Abstract

The title compound, C_37_H_52_O_4_S, was obtained by the reaction of 6,6′-(ethane-1,1-di­yl)bis­(2,4-di-*tert*-butyl­phenol) and 4-methyl­benzene-1-sulfonyl chloride. The mol­ecular conformation is stabilized by an intra­molecular O—H⋯O hydrogen bond. Two of the *tert*-butyl groups are disordered over two sets of sites with occupancies 0.530 (15)/0.470 (15) and 0.615 (11)/0.385 (11).

## Related literature

For the polymerization of cyclic esters, see: Endo *et al.* (1987 ▶); Wu *et al.* (2006 ▶).
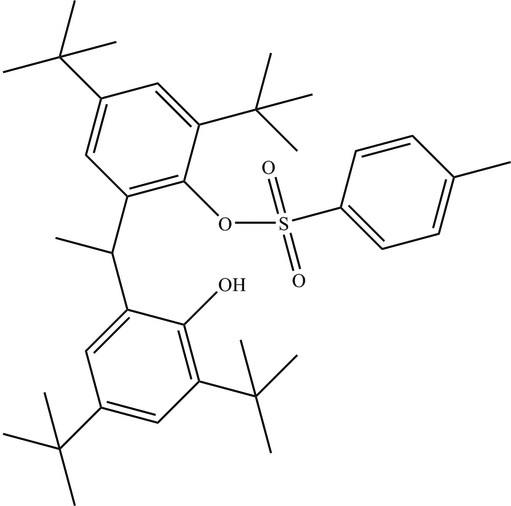


## Experimental

### 

#### Crystal data

C_37_H_52_O_4_S
*M* *_r_* = 592.85
Monoclinic, 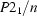

*a* = 13.893 (2) Å
*b* = 15.760 (2) Å
*c* = 17.525 (3) Å
β = 107.262 (3)°
*V* = 3664.3 (10) Å^3^
*Z* = 4
Mo *K*α radiation
μ = 0.12 mm^−1^
*T* = 298 (2) K
0.32 × 0.28 × 0.25 mm

#### Data collection

Bruker SMART 1K CCD area-detector diffractometer
Absorption correction: multi-scan (*SADABS*; Sheldrick, 2002 ▶) *T*_min_ = 0.962, *T*_max_ = 0.970
18079 measured reflections
6422 independent reflections
4281 reflections with *I* > 2σ(*I*)
*R*_int_ = 0.033

#### Refinement

*R*[*F* ^2^ > 2σ(*F* ^2^)] = 0.049
*wR*(*F* ^2^) = 0.157
*S* = 1.06
6422 reflections
435 parameters
H-atom parameters constrained
Δρ_max_ = 0.19 e Å^−3^
Δρ_min_ = −0.28 e Å^−3^

### 

Data collection: *SMART* (Bruker, 1997 ▶); cell refinement: *SAINT* (Bruker, 1997 ▶); data reduction: *SAINT*; program(s) used to solve structure: *SHELXS97* (Sheldrick, 2008 ▶); program(s) used to refine structure: *SHELXL97* (Sheldrick, 2008 ▶); molecular graphics: *SHELXTL* (Sheldrick, 2008 ▶); software used to prepare material for publication: *SHELXTL*.

## Supplementary Material

Crystal structure: contains datablocks I, global. DOI: 10.1107/S1600536808042323/bt2830sup1.cif

Structure factors: contains datablocks I. DOI: 10.1107/S1600536808042323/bt2830Isup2.hkl

Additional supplementary materials:  crystallographic information; 3D view; checkCIF report

## Figures and Tables

**Table 1:** Hydrogen-bond geometry (Å, °)

*D*—H⋯*A*	*D*—H	H⋯*A*	*D*⋯*A*	*D*—H⋯*A*
O1—H1*A*⋯O4	0.82	2.30	3.036 (2)	150

**Fig. 1.**
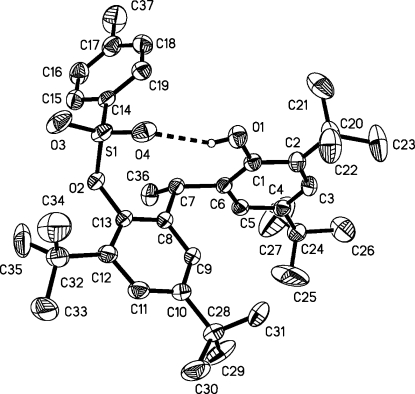
The structure of the title compound showing displacement ellipsoids drawn at the 30% probability level.

**Fig. 2.**
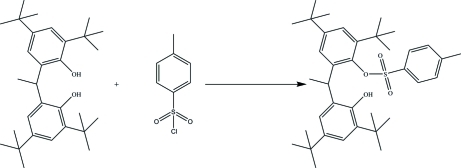
The formation of the title compound.

## supplementary crystallographic information

### Comment

In past decades, significant advance have been made in polymerization of cyclic esters, such as poly(e-caprolactone) (Endo *et al.*, 1987) or poly(lactide) (Wu *et al.*, 2006). A particularly convenient method for the synthesis of polylactides is the ring-opening polymerization (ROP) of lactides. Due to the advantages of well controlled molecular weight and low polydispersity, many metal complexes have been used. In the present study, we report a compound, which is a potential ligand for investigation of ring-opening polymerization of lactides. The bond lengths and angles are within normal ranges. There is a intramolecular hydrogen bond.

### Experimental

6,6'-(ethane-1,1-diyl)bis(2,4-di-*tert*-butylphenol) (4.38 g, 10 mmol) and triethylamine (14 mL, 100 mmol) were dissolved in 100 ml of dichloromethane. 4-Methylbenzene-1-sulfonyl chloride (2.09 g, 11 mmol) in dichloromethane (20 ml) was added dropwise into the above solution at 0 °C for about 1 h. The resulting mixture was then stirred for 24 h while the temperature was increased to room temperature. The solution was filtered, and the filtrate was washed with 50 ml of water three times. The dichloromethane layer was collected and dried over anhydrous MgSO_4_ and filtered through Celite again to remove MgSO_4_. The resulting filtrate was then dried under vacuum, and the residue was recrystallized by slow cooling of a acetonitrile solution.

### Refinement

All H atoms were placed in calculated positions and refined using a riding model, with d(O—H)=0.82Å and *U*_iso_(H)=1.2U_eq_(O), d(C—H)=0.93Å and *U*_iso_(H)=1.2U_eq_(C) for C_aromatic_, d(C—H)=0.97Å and *U*_iso_(H)=1.2U_eq_(C) for methylene groups, and d(C—H)=0.96Å and *U*_iso_(H)=1.5U_eq_(C) for methyl groups. Two *tert*-butyl groups are disordered over two sites, with a site occupation factors of 0.530 (15) and 0.615 (11) for the major occupied site, respectively.

### Crystal data

**Table:**

C_37_H_52_O_4_S	*F*(000) = 1288
*M**_r_* = 592.85	*D*_x_ = 1.075 Mg m^−^^3^
Monoclinic, *P*2_1_/*n*	Mo *K*α radiation, λ = 0.71073 Å
Hall symbol: -P 2yn	Cell parameters from 20154 reflections
*a* = 13.893 (2) Å	θ = 2.2–25.5°
*b* = 15.760 (2) Å	µ = 0.12 mm^−^^1^
*c* = 17.525 (3) Å	*T* = 298 K
β = 107.262 (3)°	Block, colourless
*V* = 3664.3 (10) Å^3^	0.32 × 0.28 × 0.25 mm
*Z* = 4	

### Data collection

**Table:**

Bruker SMART 1K CCD area-detector diffractometer	6422 independent reflections
Radiation source: fine-focus sealed tube	4281 reflections with *I* > 2σ(*I*)
graphite	*R*_int_ = 0.033
φ and ω scans	θ_max_ = 25.0°, θ_min_ = 2.0°
Absorption correction: multi-scan (*SADABS*; Sheldrick, 2002)	*h* = −16→15
*T*_min_ = 0.962, *T*_max_ = 0.970	*k* = −18→11
18079 measured reflections	*l* = −20→20

### Refinement

**Table:**

Refinement on *F*^2^	Primary atom site location: structure-invariant direct methods
Least-squares matrix: full	Secondary atom site location: difference Fourier map
*R*[*F*^2^ > 2σ(*F*^2^)] = 0.049	Hydrogen site location: inferred from neighbouring sites
*wR*(*F*^2^) = 0.157	H-atom parameters constrained
*S* = 1.06	*w* = 1/[σ^2^(*F*_o_^2^) + (0.0922*P*)^2^] where *P* = (*F*_o_^2^ + 2*F*_c_^2^)/3
6422 reflections	(Δ/σ)_max_ < 0.001
435 parameters	Δρ_max_ = 0.19 e Å^−^^3^
0 restraints	Δρ_min_ = −0.28 e Å^−^^3^

### Special details

**Table:**

Geometry. All e.s.d.'s (except the e.s.d. in the dihedral angle between two l.s. planes) are estimated using the full covariance matrix. The cell e.s.d.'s are taken into account individually in the estimation of e.s.d.'s in distances, angles and torsion angles; correlations between e.s.d.'s in cell parameters are only used when they are defined by crystal symmetry. An approximate (isotropic) treatment of cell e.s.d.'s is used for estimating e.s.d.'s involving l.s. planes.
Refinement. Refinement of *F*^2^ against ALL reflections. The weighted *R*-factor *wR* and goodness of fit *S* are based on *F*^2^, conventional *R*-factors *R* are based on *F*, with *F* set to zero for negative *F*^2^. The threshold expression of *F*^2^ > σ(*F*^2^) is used only for calculating *R*-factors(gt) *etc*. and is not relevant to the choice of reflections for refinement. *R*-factors based on *F*^2^ are statistically about twice as large as those based on *F*, and *R*- factors based on ALL data will be even larger.

### Fractional atomic coordinates and isotropic or equivalent isotropic displacement parameters (Å^2^)

**Table:**

	*x*	*y*	*z*	*U*_iso_*/*U*_eq_	Occ. (<1)
S1	−0.12768 (4)	0.86568 (4)	0.82030 (3)	0.0582 (2)	
O1	−0.03328 (10)	0.65695 (9)	0.72763 (9)	0.0660 (4)	
H1A	−0.0523	0.7046	0.7351	0.099*	
O2	−0.08263 (10)	0.92862 (9)	0.76869 (8)	0.0560 (4)	
O3	−0.19479 (13)	0.91394 (13)	0.84952 (9)	0.0892 (6)	
O4	−0.16166 (11)	0.79066 (10)	0.77557 (8)	0.0706 (5)	
C1	0.04851 (14)	0.66320 (13)	0.69863 (11)	0.0495 (5)	
C2	0.08001 (15)	0.58978 (13)	0.66879 (13)	0.0565 (5)	
C3	0.16357 (16)	0.59769 (14)	0.64116 (13)	0.0604 (6)	
H3B	0.1858	0.5496	0.6207	0.072*	
C4	0.21551 (15)	0.67205 (14)	0.64218 (12)	0.0544 (5)	
C5	0.18127 (15)	0.74257 (13)	0.67293 (11)	0.0519 (5)	
H5A	0.2153	0.7937	0.6748	0.062*	
C6	0.09822 (14)	0.73996 (12)	0.70110 (10)	0.0456 (5)	
C7	0.05885 (14)	0.81936 (12)	0.73091 (11)	0.0473 (5)	
H7A	0.0331	0.8020	0.7749	0.057*	
C8	−0.02946 (14)	0.85829 (12)	0.66655 (11)	0.0446 (5)	
C9	−0.04110 (15)	0.84412 (12)	0.58635 (11)	0.0481 (5)	
H9A	0.0024	0.8065	0.5724	0.058*	
C10	−0.11499 (16)	0.88377 (13)	0.52618 (12)	0.0527 (5)	
C11	−0.17554 (16)	0.94102 (14)	0.54969 (12)	0.0600 (6)	
H11A	−0.2250	0.9687	0.5099	0.072*	
C12	−0.16794 (16)	0.96036 (14)	0.62890 (13)	0.0583 (6)	
C13	−0.09589 (14)	0.91427 (12)	0.68549 (11)	0.0482 (5)	
C14	−0.02230 (15)	0.84260 (14)	0.90080 (11)	0.0496 (5)	
C15	0.03200 (17)	0.90683 (15)	0.94664 (13)	0.0648 (6)	
H15A	0.0149	0.9632	0.9336	0.078*	
C16	0.11210 (19)	0.88714 (17)	1.01221 (14)	0.0746 (7)	
H16A	0.1497	0.9308	1.0425	0.089*	
C17	0.13759 (18)	0.80494 (18)	1.03374 (13)	0.0723 (7)	
C18	0.08153 (19)	0.74164 (16)	0.98730 (14)	0.0748 (7)	
H18A	0.0976	0.6853	1.0012	0.090*	
C19	0.00230 (17)	0.75959 (15)	0.92091 (13)	0.0638 (6)	
H19A	−0.0343	0.7159	0.8899	0.077*	
C20	0.02661 (19)	0.50438 (15)	0.66815 (19)	0.0798 (8)	
C21	0.0425 (2)	0.47584 (19)	0.7540 (2)	0.1130 (11)	
H21A	0.0166	0.5182	0.7820	0.169*	
H21B	0.1132	0.4681	0.7799	0.169*	
H21C	0.0078	0.4232	0.7542	0.169*	
C22	−0.0861 (2)	0.51147 (19)	0.6233 (2)	0.1080 (11)	
H22A	−0.1164	0.5532	0.6488	0.162*	
H22B	−0.1179	0.4575	0.6239	0.162*	
H22C	−0.0944	0.5281	0.5690	0.162*	
C23	0.0693 (2)	0.43513 (19)	0.6248 (3)	0.1288 (14)	
H23A	0.1398	0.4277	0.6515	0.193*	
H23B	0.0600	0.4520	0.5705	0.193*	
H23C	0.0345	0.3827	0.6256	0.193*	
C24	0.30546 (19)	0.67786 (16)	0.60915 (15)	0.0708 (7)	
C25	0.2779 (13)	0.7341 (10)	0.5397 (10)	0.201 (11)	0.530 (15)
H25A	0.2208	0.7110	0.4998	0.402*	0.530 (15)
H25B	0.3337	0.7391	0.5183	0.402*	0.530 (15)
H25C	0.2611	0.7891	0.5555	0.402*	0.530 (15)
C26	0.3269 (9)	0.5914 (4)	0.5741 (8)	0.113 (4)	0.530 (15)
H26A	0.2690	0.5748	0.5312	0.227*	0.530 (15)
H26B	0.3409	0.5489	0.6152	0.227*	0.530 (15)
H26C	0.3840	0.5972	0.5543	0.227*	0.530 (15)
C27	0.3962 (6)	0.7038 (15)	0.6763 (8)	0.184 (10)	0.530 (15)
H27A	0.4178	0.6571	0.7125	0.369*	0.530 (15)
H27B	0.3790	0.7510	0.7044	0.369*	0.530 (15)
H27C	0.4497	0.7200	0.6550	0.369*	0.530 (15)
C25'	0.2691 (10)	0.6693 (12)	0.5187 (6)	0.167 (10)	0.470 (15)
H25D	0.2003	0.6505	0.5022	0.335*	0.470 (15)
H25E	0.3103	0.6287	0.5020	0.335*	0.470 (15)
H25F	0.2738	0.7233	0.4947	0.335*	0.470 (15)
C26'	0.3858 (8)	0.6148 (10)	0.6495 (12)	0.149 (9)	0.470 (15)
H26D	0.3668	0.5594	0.6273	0.298*	0.470 (15)
H26E	0.3934	0.6138	0.7057	0.298*	0.470 (15)
H26F	0.4486	0.6310	0.6412	0.298*	0.470 (15)
C27'	0.3555 (8)	0.7668 (5)	0.6201 (8)	0.104 (4)	0.470 (15)
H27D	0.3206	0.8028	0.5764	0.209*	0.470 (15)
H27E	0.4247	0.7616	0.6213	0.209*	0.470 (15)
H27F	0.3520	0.7912	0.6694	0.209*	0.470 (15)
C28	−0.12671 (18)	0.86591 (15)	0.43838 (12)	0.0649 (6)	
C29	−0.0325 (5)	0.8900 (7)	0.4209 (3)	0.121 (4)	0.615 (11)
H29A	−0.0310	0.9504	0.4142	0.242*	0.615 (11)
H29B	−0.0298	0.8625	0.3727	0.242*	0.615 (11)
H29C	0.0244	0.8729	0.4644	0.242*	0.615 (11)
C30	−0.2182 (5)	0.9135 (5)	0.3822 (3)	0.112 (4)	0.615 (11)
H30D	−0.2298	0.8937	0.3284	0.224*	0.615 (11)
H30A	−0.2045	0.9733	0.3845	0.224*	0.615 (11)
H30B	−0.2769	0.9030	0.3990	0.224*	0.615 (11)
C31	−0.1505 (8)	0.7724 (4)	0.4254 (4)	0.115 (3)	0.615 (11)
H31A	−0.2184	0.7621	0.4260	0.230*	0.615 (11)
H31B	−0.1047	0.7402	0.4672	0.230*	0.615 (11)
H31C	−0.1435	0.7554	0.3747	0.230*	0.615 (11)
C29'	−0.1198 (11)	0.9495 (6)	0.3951 (5)	0.113 (5)	0.385 (11)
H29D	−0.1734	0.9866	0.3979	0.225*	0.385 (11)
H29E	−0.1254	0.9379	0.3402	0.225*	0.385 (11)
H29F	−0.0561	0.9762	0.4203	0.225*	0.385 (11)
C30'	−0.2196 (15)	0.8198 (14)	0.3989 (6)	0.195 (12)	0.385 (11)
H30E	−0.2764	0.8506	0.4052	0.389*	0.385 (11)
H30F	−0.2172	0.7646	0.4226	0.389*	0.385 (11)
H30G	−0.2262	0.8138	0.3431	0.389*	0.385 (11)
C31'	−0.0357 (13)	0.8070 (12)	0.4275 (5)	0.145 (7)	0.385 (11)
H31D	−0.0493	0.7486	0.4360	0.289*	0.385 (11)
H31E	0.0266	0.8237	0.4657	0.289*	0.385 (11)
H31F	−0.0305	0.8139	0.3745	0.289*	0.385 (11)
C32	−0.2354 (2)	1.02994 (19)	0.64732 (17)	0.0883 (9)	
C33	−0.2761 (3)	1.0877 (3)	0.5742 (2)	0.165 (2)	
H33A	−0.3177	1.1311	0.5864	0.247*	
H33B	−0.3153	1.0546	0.5298	0.247*	
H33C	−0.2207	1.1135	0.5607	0.247*	
C34	−0.3234 (2)	0.9887 (3)	0.6664 (2)	0.1381 (15)	
H34A	−0.3659	1.0318	0.6780	0.207*	
H34B	−0.2991	0.9523	0.7119	0.207*	
H34C	−0.3614	0.9559	0.6213	0.207*	
C35	−0.1797 (3)	1.0870 (2)	0.7152 (2)	0.1520 (17)	
H35A	−0.2250	1.1293	0.7241	0.228*	
H35B	−0.1249	1.1142	0.7020	0.228*	
H35C	−0.1537	1.0538	0.7629	0.228*	
C36	0.13897 (16)	0.88649 (14)	0.76382 (14)	0.0673 (6)	
H36A	0.1091	0.9345	0.7818	0.101*	
H36B	0.1673	0.9041	0.7226	0.101*	
H36C	0.1912	0.8633	0.8078	0.101*	
C37	0.2241 (2)	0.7836 (2)	1.10578 (17)	0.1190 (12)	
H37A	0.2303	0.7231	1.1114	0.178*	
H37B	0.2122	0.8074	1.1526	0.178*	
H37C	0.2853	0.8067	1.0995	0.178*	

### Atomic displacement parameters (Å^2^)

**Table:**

	*U*^11^	*U*^22^	*U*^33^	*U*^12^	*U*^13^	*U*^23^
S1	0.0529 (3)	0.0830 (5)	0.0432 (3)	0.0073 (3)	0.0212 (2)	0.0026 (3)
O1	0.0584 (9)	0.0631 (10)	0.0872 (11)	−0.0002 (7)	0.0381 (8)	−0.0085 (8)
O2	0.0696 (9)	0.0589 (9)	0.0458 (7)	0.0095 (7)	0.0269 (7)	−0.0021 (6)
O3	0.0729 (10)	0.1432 (17)	0.0623 (10)	0.0428 (10)	0.0366 (9)	0.0113 (10)
O4	0.0677 (10)	0.0892 (12)	0.0520 (9)	−0.0206 (8)	0.0135 (8)	−0.0033 (8)
C1	0.0447 (11)	0.0573 (13)	0.0483 (11)	0.0084 (9)	0.0165 (9)	0.0009 (9)
C2	0.0515 (12)	0.0550 (13)	0.0647 (13)	0.0076 (10)	0.0199 (10)	−0.0038 (10)
C3	0.0670 (14)	0.0532 (14)	0.0674 (14)	0.0132 (11)	0.0298 (12)	−0.0062 (11)
C4	0.0554 (12)	0.0608 (14)	0.0536 (12)	0.0128 (10)	0.0262 (10)	0.0066 (10)
C5	0.0558 (12)	0.0528 (13)	0.0522 (12)	0.0065 (9)	0.0237 (10)	0.0053 (9)
C6	0.0477 (10)	0.0506 (12)	0.0398 (10)	0.0088 (9)	0.0148 (9)	0.0015 (8)
C7	0.0511 (11)	0.0501 (12)	0.0420 (10)	0.0069 (9)	0.0158 (9)	−0.0015 (9)
C8	0.0483 (11)	0.0445 (11)	0.0437 (11)	0.0027 (8)	0.0176 (9)	0.0000 (8)
C9	0.0552 (12)	0.0479 (12)	0.0442 (11)	0.0043 (9)	0.0192 (9)	−0.0031 (9)
C10	0.0602 (12)	0.0543 (13)	0.0459 (11)	−0.0002 (10)	0.0193 (10)	0.0049 (9)
C11	0.0600 (13)	0.0702 (15)	0.0490 (12)	0.0160 (11)	0.0148 (10)	0.0140 (10)
C12	0.0623 (13)	0.0631 (14)	0.0562 (13)	0.0179 (11)	0.0278 (11)	0.0134 (10)
C13	0.0570 (12)	0.0524 (12)	0.0400 (10)	0.0059 (9)	0.0215 (9)	−0.0001 (9)
C14	0.0541 (11)	0.0599 (14)	0.0394 (10)	0.0031 (10)	0.0212 (9)	−0.0012 (9)
C15	0.0745 (15)	0.0609 (15)	0.0578 (13)	0.0039 (12)	0.0177 (12)	−0.0068 (11)
C16	0.0768 (16)	0.0788 (18)	0.0601 (15)	−0.0001 (13)	0.0081 (13)	−0.0181 (13)
C17	0.0730 (15)	0.0880 (19)	0.0522 (13)	0.0146 (14)	0.0129 (12)	−0.0052 (13)
C18	0.0925 (18)	0.0661 (16)	0.0597 (15)	0.0127 (14)	0.0131 (14)	0.0066 (12)
C19	0.0744 (15)	0.0620 (15)	0.0535 (13)	−0.0027 (11)	0.0168 (12)	−0.0002 (11)
C20	0.0681 (15)	0.0557 (15)	0.125 (2)	−0.0031 (11)	0.0424 (16)	−0.0167 (15)
C21	0.110 (2)	0.075 (2)	0.168 (3)	−0.0019 (17)	0.062 (2)	0.028 (2)
C22	0.0727 (17)	0.094 (2)	0.159 (3)	−0.0192 (15)	0.0376 (19)	−0.045 (2)
C23	0.110 (2)	0.070 (2)	0.226 (4)	−0.0109 (17)	0.079 (3)	−0.055 (2)
C24	0.0775 (16)	0.0733 (17)	0.0801 (17)	0.0113 (13)	0.0518 (14)	0.0063 (13)
C25	0.28 (2)	0.192 (13)	0.220 (19)	0.124 (14)	0.219 (18)	0.136 (13)
C26	0.130 (7)	0.097 (5)	0.157 (9)	0.036 (5)	0.111 (7)	0.021 (5)
C27	0.069 (5)	0.35 (3)	0.165 (10)	−0.054 (10)	0.075 (6)	−0.077 (15)
C25'	0.164 (12)	0.27 (2)	0.106 (8)	−0.091 (15)	0.100 (9)	−0.078 (12)
C26'	0.094 (7)	0.170 (11)	0.22 (2)	0.069 (8)	0.100 (11)	0.106 (14)
C27'	0.109 (7)	0.101 (6)	0.141 (10)	−0.029 (5)	0.095 (7)	−0.023 (5)
C28	0.0802 (16)	0.0712 (16)	0.0409 (12)	0.0019 (12)	0.0142 (11)	−0.0007 (10)
C29	0.114 (5)	0.210 (12)	0.058 (3)	−0.048 (7)	0.054 (3)	−0.026 (5)
C30	0.126 (6)	0.154 (7)	0.045 (2)	0.044 (5)	0.007 (3)	0.016 (3)
C31	0.194 (9)	0.091 (4)	0.052 (3)	−0.003 (4)	0.024 (4)	−0.030 (3)
C29'	0.181 (17)	0.108 (7)	0.062 (5)	−0.003 (7)	0.056 (7)	0.017 (4)
C30'	0.233 (18)	0.28 (3)	0.056 (6)	−0.18 (2)	0.016 (9)	−0.015 (11)
C31'	0.225 (15)	0.164 (13)	0.063 (5)	0.077 (12)	0.069 (7)	0.002 (6)
C32	0.099 (2)	0.100 (2)	0.0807 (18)	0.0581 (17)	0.0498 (16)	0.0325 (16)
C33	0.230 (5)	0.167 (4)	0.133 (3)	0.152 (4)	0.110 (3)	0.081 (3)
C34	0.095 (2)	0.196 (4)	0.145 (3)	0.067 (2)	0.069 (2)	0.044 (3)
C35	0.209 (4)	0.106 (3)	0.141 (3)	0.082 (3)	0.053 (3)	−0.027 (2)
C36	0.0584 (13)	0.0668 (15)	0.0723 (15)	0.0026 (11)	0.0125 (12)	−0.0159 (12)
C37	0.107 (2)	0.146 (3)	0.0741 (19)	0.041 (2)	−0.0190 (17)	−0.0131 (19)

### Geometric parameters (Å, °)

**Table:**

S1—O3	1.4118 (15)	C25—H25A	0.9600
S1—O4	1.4197 (16)	C25—H25B	0.9600
S1—O2	1.5905 (14)	C25—H25C	0.9600
S1—C14	1.744 (2)	C26—H26A	0.9600
O1—C1	1.379 (2)	C26—H26B	0.9600
O1—H1A	0.8200	C26—H26C	0.9600
O2—C13	1.432 (2)	C27—H27A	0.9600
C1—C6	1.387 (3)	C27—H27B	0.9600
C1—C2	1.393 (3)	C27—H27C	0.9600
C2—C3	1.390 (3)	C25'—H25D	0.9600
C2—C20	1.535 (3)	C25'—H25E	0.9600
C3—C4	1.374 (3)	C25'—H25F	0.9600
C3—H3B	0.9300	C26'—H26D	0.9600
C4—C5	1.380 (3)	C26'—H26E	0.9600
C4—C24	1.528 (3)	C26'—H26F	0.9600
C5—C6	1.384 (3)	C27'—H27D	0.9600
C5—H5A	0.9300	C27'—H27E	0.9600
C6—C7	1.519 (3)	C27'—H27F	0.9600
C7—C36	1.520 (3)	C28—C30'	1.464 (11)
C7—C8	1.527 (3)	C28—C29	1.480 (5)
C7—H7A	0.9800	C28—C31	1.513 (7)
C8—C9	1.384 (3)	C28—C29'	1.537 (8)
C8—C13	1.386 (3)	C28—C30	1.551 (5)
C9—C10	1.383 (3)	C28—C31'	1.625 (11)
C9—H9A	0.9300	C29—H29A	0.9600
C10—C11	1.377 (3)	C29—H29B	0.9600
C10—C28	1.525 (3)	C29—H29C	0.9600
C11—C12	1.394 (3)	C30—H30D	0.9600
C11—H11A	0.9300	C30—H30A	0.9600
C12—C13	1.387 (3)	C30—H30B	0.9600
C12—C32	1.538 (3)	C31—H31A	0.9600
C14—C15	1.371 (3)	C31—H31B	0.9600
C14—C19	1.371 (3)	C31—H31C	0.9600
C15—C16	1.377 (3)	C29'—H29D	0.9600
C15—H15A	0.9300	C29'—H29E	0.9600
C16—C17	1.366 (3)	C29'—H29F	0.9600
C16—H16A	0.9300	C30'—H30E	0.9600
C17—C18	1.374 (3)	C30'—H30F	0.9600
C17—C37	1.501 (3)	C30'—H30G	0.9600
C18—C19	1.373 (3)	C31'—H31D	0.9600
C18—H18A	0.9300	C31'—H31E	0.9600
C19—H19A	0.9300	C31'—H31F	0.9600
C20—C21	1.522 (4)	C32—C34	1.507 (4)
C20—C22	1.533 (4)	C32—C35	1.509 (5)
C20—C23	1.544 (4)	C32—C33	1.537 (4)
C21—H21A	0.9600	C33—H33A	0.9600
C21—H21B	0.9600	C33—H33B	0.9600
C21—H21C	0.9600	C33—H33C	0.9600
C22—H22A	0.9600	C34—H34A	0.9600
C22—H22B	0.9600	C34—H34B	0.9600
C22—H22C	0.9600	C34—H34C	0.9600
C23—H23A	0.9600	C35—H35A	0.9600
C23—H23B	0.9600	C35—H35B	0.9600
C23—H23C	0.9600	C35—H35C	0.9600
C24—C25	1.461 (9)	C36—H36A	0.9600
C24—C27	1.504 (9)	C36—H36B	0.9600
C24—C26'	1.505 (8)	C36—H36C	0.9600
C24—C25'	1.521 (10)	C37—H37A	0.9600
C24—C27'	1.552 (7)	C37—H37B	0.9600
C24—C26	1.560 (7)	C37—H37C	0.9600
			
O3—S1—O4	120.11 (11)	C24—C27—H27A	109.5
O3—S1—O2	106.49 (10)	C24—C27—H27B	109.5
O4—S1—O2	109.14 (8)	H27A—C27—H27B	109.5
O3—S1—C14	107.78 (9)	C24—C27—H27C	109.5
O4—S1—C14	109.56 (10)	H27A—C27—H27C	109.5
O2—S1—C14	102.28 (9)	H27B—C27—H27C	109.5
C1—O1—H1A	109.5	C24—C25'—H25D	109.5
C13—O2—S1	121.80 (12)	C24—C25'—H25E	109.5
O1—C1—C6	120.51 (17)	H25D—C25'—H25E	109.5
O1—C1—C2	117.64 (18)	C24—C25'—H25F	109.5
C6—C1—C2	121.83 (18)	H25D—C25'—H25F	109.5
C3—C2—C1	116.4 (2)	H25E—C25'—H25F	109.5
C3—C2—C20	121.87 (19)	C24—C26'—H26D	109.5
C1—C2—C20	121.75 (19)	C24—C26'—H26E	109.5
C4—C3—C2	124.19 (19)	H26D—C26'—H26E	109.5
C4—C3—H3B	117.9	C24—C26'—H26F	109.5
C2—C3—H3B	117.9	H26D—C26'—H26F	109.5
C3—C4—C5	116.85 (18)	H26E—C26'—H26F	109.5
C3—C4—C24	122.12 (19)	C24—C27'—H27D	109.5
C5—C4—C24	121.0 (2)	C24—C27'—H27E	109.5
C4—C5—C6	122.4 (2)	H27D—C27'—H27E	109.5
C4—C5—H5A	118.8	C24—C27'—H27F	109.5
C6—C5—H5A	118.8	H27D—C27'—H27F	109.5
C5—C6—C1	118.35 (18)	H27E—C27'—H27F	109.5
C5—C6—C7	121.44 (18)	C30'—C28—C29	137.7 (6)
C1—C6—C7	120.16 (16)	C30'—C28—C31	47.4 (9)
C6—C7—C36	113.96 (16)	C29—C28—C31	112.7 (5)
C6—C7—C8	111.68 (15)	C30'—C28—C10	112.5 (5)
C36—C7—C8	109.75 (17)	C29—C28—C10	109.0 (3)
C6—C7—H7A	107.0	C31—C28—C10	106.6 (3)
C36—C7—H7A	107.0	C30'—C28—C29'	111.6 (9)
C8—C7—H7A	107.0	C29—C28—C29'	59.2 (5)
C9—C8—C13	117.40 (17)	C31—C28—C29'	143.5 (4)
C9—C8—C7	120.74 (16)	C10—C28—C29'	109.6 (3)
C13—C8—C7	121.66 (16)	C30'—C28—C30	59.9 (9)
C10—C9—C8	122.53 (18)	C29—C28—C30	110.7 (4)
C10—C9—H9A	118.7	C31—C28—C30	105.9 (4)
C8—C9—H9A	118.7	C10—C28—C30	111.9 (2)
C11—C10—C9	116.66 (18)	C29'—C28—C30	55.4 (5)
C11—C10—C28	122.07 (19)	C30'—C28—C31'	105.6 (10)
C9—C10—C28	121.25 (18)	C29—C28—C31'	49.9 (6)
C10—C11—C12	124.64 (19)	C31—C28—C31'	64.4 (7)
C10—C11—H11A	117.7	C10—C28—C31'	111.7 (4)
C12—C11—H11A	117.7	C29'—C28—C31'	105.5 (7)
C13—C12—C11	115.01 (18)	C30—C28—C31'	136.2 (4)
C13—C12—C32	125.37 (19)	C28—C29—H29A	109.5
C11—C12—C32	119.61 (19)	C28—C29—H29B	109.5
C8—C13—C12	123.54 (17)	H29A—C29—H29B	109.5
C8—C13—O2	116.77 (16)	C28—C29—H29C	109.5
C12—C13—O2	119.52 (16)	H29A—C29—H29C	109.5
C15—C14—C19	120.1 (2)	H29B—C29—H29C	109.5
C15—C14—S1	120.26 (17)	C28—C30—H30D	109.5
C19—C14—S1	119.49 (16)	C28—C30—H30A	109.5
C14—C15—C16	119.4 (2)	H30D—C30—H30A	109.5
C14—C15—H15A	120.3	C28—C30—H30B	109.5
C16—C15—H15A	120.3	H30D—C30—H30B	109.5
C17—C16—C15	121.6 (2)	H30A—C30—H30B	109.5
C17—C16—H16A	119.2	C28—C31—H31A	109.5
C15—C16—H16A	119.2	C28—C31—H31B	109.5
C16—C17—C18	118.0 (2)	H31A—C31—H31B	109.5
C16—C17—C37	121.5 (3)	C28—C31—H31C	109.5
C18—C17—C37	120.5 (3)	H31A—C31—H31C	109.5
C19—C18—C17	121.6 (2)	H31B—C31—H31C	109.5
C19—C18—H18A	119.2	C28—C29'—H29D	109.5
C17—C18—H18A	119.2	C28—C29'—H29E	109.5
C14—C19—C18	119.4 (2)	H29D—C29'—H29E	109.5
C14—C19—H19A	120.3	C28—C29'—H29F	109.5
C18—C19—H19A	120.3	H29D—C29'—H29F	109.5
C21—C20—C22	110.8 (3)	H29E—C29'—H29F	109.5
C21—C20—C2	108.9 (2)	C28—C30'—H30E	109.5
C22—C20—C2	110.9 (2)	C28—C30'—H30F	109.5
C21—C20—C23	108.2 (3)	H30E—C30'—H30F	109.5
C22—C20—C23	106.4 (2)	C28—C30'—H30G	109.5
C2—C20—C23	111.6 (2)	H30E—C30'—H30G	109.5
C20—C21—H21A	109.5	H30F—C30'—H30G	109.5
C20—C21—H21B	109.5	C28—C31'—H31D	109.5
H21A—C21—H21B	109.5	C28—C31'—H31E	109.5
C20—C21—H21C	109.5	H31D—C31'—H31E	109.5
H21A—C21—H21C	109.5	C28—C31'—H31F	109.5
H21B—C21—H21C	109.5	H31D—C31'—H31F	109.5
C20—C22—H22A	109.5	H31E—C31'—H31F	109.5
C20—C22—H22B	109.5	C34—C32—C35	110.0 (3)
H22A—C22—H22B	109.5	C34—C32—C33	108.6 (3)
C20—C22—H22C	109.5	C35—C32—C33	106.3 (3)
H22A—C22—H22C	109.5	C34—C32—C12	108.9 (3)
H22B—C22—H22C	109.5	C35—C32—C12	112.9 (2)
C20—C23—H23A	109.5	C33—C32—C12	110.1 (2)
C20—C23—H23B	109.5	C32—C33—H33A	109.5
H23A—C23—H23B	109.5	C32—C33—H33B	109.5
C20—C23—H23C	109.5	H33A—C33—H33B	109.5
H23A—C23—H23C	109.5	C32—C33—H33C	109.5
H23B—C23—H23C	109.5	H33A—C33—H33C	109.5
C25—C24—C27	116.0 (10)	H33B—C33—H33C	109.5
C26'—C24—C25'	113.7 (9)	C32—C34—H34A	109.5
C25—C24—C4	108.1 (5)	C32—C34—H34B	109.5
C27—C24—C4	108.0 (3)	H34A—C34—H34B	109.5
C26'—C24—C4	111.0 (3)	C32—C34—H34C	109.5
C25'—C24—C4	109.5 (5)	H34A—C34—H34C	109.5
C26'—C24—C27'	107.2 (6)	H34B—C34—H34C	109.5
C25'—C24—C27'	102.0 (6)	C32—C35—H35A	109.5
C4—C24—C27'	113.2 (3)	C32—C35—H35B	109.5
C25—C24—C26	103.6 (6)	H35A—C35—H35B	109.5
C27—C24—C26	109.4 (7)	C32—C35—H35C	109.5
C4—C24—C26	111.7 (3)	H35A—C35—H35C	109.5
C27'—C24—C26	135.1 (4)	H35B—C35—H35C	109.5
C24—C25—H25A	109.5	C7—C36—H36A	109.5
C24—C25—H25B	109.5	C7—C36—H36B	109.5
H25A—C25—H25B	109.5	H36A—C36—H36B	109.5
C24—C25—H25C	109.5	C7—C36—H36C	109.5
H25A—C25—H25C	109.5	H36A—C36—H36C	109.5
H25B—C25—H25C	109.5	H36B—C36—H36C	109.5
C24—C26—H26A	109.5	C17—C37—H37A	109.5
C24—C26—H26B	109.5	C17—C37—H37B	109.5
H26A—C26—H26B	109.5	H37A—C37—H37B	109.5
C24—C26—H26C	109.5	C17—C37—H37C	109.5
H26A—C26—H26C	109.5	H37A—C37—H37C	109.5
H26B—C26—H26C	109.5	H37B—C37—H37C	109.5
			
O3—S1—O2—C13	−119.82 (15)	O4—S1—C14—C19	−12.53 (19)
O4—S1—O2—C13	11.22 (16)	O2—S1—C14—C19	−128.22 (16)
C14—S1—O2—C13	127.21 (14)	C19—C14—C15—C16	0.9 (3)
O1—C1—C2—C3	−178.99 (18)	S1—C14—C15—C16	176.94 (17)
C6—C1—C2—C3	−0.4 (3)	C14—C15—C16—C17	−1.4 (4)
O1—C1—C2—C20	−0.3 (3)	C15—C16—C17—C18	0.8 (4)
C6—C1—C2—C20	178.3 (2)	C15—C16—C17—C37	−179.3 (3)
C1—C2—C3—C4	0.4 (3)	C16—C17—C18—C19	0.2 (4)
C20—C2—C3—C4	−178.4 (2)	C37—C17—C18—C19	−179.7 (3)
C2—C3—C4—C5	0.1 (3)	C15—C14—C19—C18	0.1 (3)
C2—C3—C4—C24	−178.5 (2)	S1—C14—C19—C18	−175.96 (17)
C3—C4—C5—C6	−0.5 (3)	C17—C18—C19—C14	−0.7 (4)
C24—C4—C5—C6	178.05 (19)	C3—C2—C20—C21	112.6 (3)
C4—C5—C6—C1	0.5 (3)	C1—C2—C20—C21	−66.1 (3)
C4—C5—C6—C7	−176.95 (17)	C3—C2—C20—C22	−125.2 (2)
O1—C1—C6—C5	178.56 (17)	C1—C2—C20—C22	56.1 (3)
C2—C1—C6—C5	0.0 (3)	C3—C2—C20—C23	−6.8 (4)
O1—C1—C6—C7	−4.0 (3)	C1—C2—C20—C23	174.5 (3)
C2—C1—C6—C7	177.45 (17)	C3—C4—C24—C25	114.6 (10)
C5—C6—C7—C36	−27.5 (3)	C5—C4—C24—C25	−63.9 (10)
C1—C6—C7—C36	155.09 (19)	C3—C4—C24—C27	−119.2 (10)
C5—C6—C7—C8	97.5 (2)	C5—C4—C24—C27	62.3 (10)
C1—C6—C7—C8	−79.8 (2)	C3—C4—C24—C26'	−56.7 (10)
C6—C7—C8—C9	−24.8 (3)	C5—C4—C24—C26'	124.9 (10)
C36—C7—C8—C9	102.6 (2)	C3—C4—C24—C25'	69.7 (8)
C6—C7—C8—C13	160.55 (17)	C5—C4—C24—C25'	−108.8 (8)
C36—C7—C8—C13	−72.1 (2)	C3—C4—C24—C27'	−177.3 (7)
C13—C8—C9—C10	0.4 (3)	C5—C4—C24—C27'	4.2 (7)
C7—C8—C9—C10	−174.48 (18)	C3—C4—C24—C26	1.2 (7)
C8—C9—C10—C11	2.1 (3)	C5—C4—C24—C26	−177.3 (6)
C8—C9—C10—C28	−179.19 (19)	C11—C10—C28—C30'	−70.0 (12)
C9—C10—C11—C12	−0.8 (3)	C9—C10—C28—C30'	111.4 (12)
C28—C10—C11—C12	−179.6 (2)	C11—C10—C28—C29	118.1 (5)
C10—C11—C12—C13	−2.8 (3)	C9—C10—C28—C29	−60.6 (5)
C10—C11—C12—C32	175.9 (2)	C11—C10—C28—C31	−120.0 (5)
C9—C8—C13—C12	−4.5 (3)	C9—C10—C28—C31	61.3 (5)
C7—C8—C13—C12	170.40 (19)	C11—C10—C28—C29'	54.9 (6)
C9—C8—C13—O2	−179.71 (16)	C9—C10—C28—C29'	−123.8 (6)
C7—C8—C13—O2	−4.9 (3)	C11—C10—C28—C30	−4.7 (5)
C11—C12—C13—C8	5.5 (3)	C9—C10—C28—C30	176.6 (4)
C32—C12—C13—C8	−173.1 (2)	C11—C10—C28—C31'	171.4 (9)
C11—C12—C13—O2	−179.38 (18)	C9—C10—C28—C31'	−7.2 (9)
C32—C12—C13—O2	2.0 (3)	C13—C12—C32—C34	−81.7 (3)
S1—O2—C13—C8	−82.7 (2)	C11—C12—C32—C34	99.8 (3)
S1—O2—C13—C12	101.8 (2)	C13—C12—C32—C35	40.8 (4)
O3—S1—C14—C15	−56.30 (19)	C11—C12—C32—C35	−137.8 (3)
O4—S1—C14—C15	171.40 (16)	C13—C12—C32—C33	159.3 (3)
O2—S1—C14—C15	55.71 (17)	C11—C12—C32—C33	−19.2 (4)
O3—S1—C14—C19	119.77 (18)		

### Hydrogen-bond geometry (Å, °)

**Table:**

*D*—H···*A*	*D*—H	H···*A*	*D*···*A*	*D*—H···*A*
O1—H1A···O4	0.82	2.30	3.036 (2)	150
